# Children Only 3 Years Old Can Succeed at Conditional “If, Then” Reasoning, Much Earlier Than Anyone Had Thought Possible

**DOI:** 10.3389/fpsyg.2020.571891

**Published:** 2021-01-06

**Authors:** Daphne S. Ling, Cole D. Wong, Adele Diamond

**Affiliations:** Developmental Cognitive Neuroscience Program, Department of Psychiatry, The University of British Columbia, Vancouver, BC, Canada

**Keywords:** pull, preschoolers, young children, conceptual understanding, conditional associative learning, dimensional change card sort

## Abstract

That conditional, if-then reasoning does not emerge until 4–5 years has long been accepted. Here we show that children barely 3 years old can do conditional reasoning. All that was needed was a superficial change to the stimuli: When color was a property of the shapes (line drawings of a star and truck) rather than of the background (as in all past conditional discrimination [CD] testing), 3-year-olds could succeed. Three-year-olds do not seem to use color to inform them which shape is correct unless color is a property of the shapes themselves. While CD requires integrating color and shape information, the dimensional change card sort (DCCS) task requires keeping those dimension cognitively separate – inhibiting attention to one (e.g., shape) when sorting by the other (e.g., color). For DCCS, a superficial change to the stimuli that is the inverse of what helps on CD enables 3-year-olds to succeed when normally they do not until $∼4+\frac{1}{2}$ years. As we and others have previously shown, 3-year-olds can succeed at DCCS when color is a property of the background (e.g., a white truck on a red background), instead of a property of the stimulus (e.g., a red truck on a white background, as in standard DCCS). Our findings on CD and DCCS suggest that scaffolding preschoolers’ emerging conceptual skills by changing the way stimuli look (perceptual bootstrapping) enables 3-year-olds to demonstrate reasoning abilities long thought beyond their grasp. Evidently, children of 3 years have difficulty mentally separating dimensions (e.g., color and shape) of the same object and difficulty mentally integrating dimensions not part of the same object. Our present CD findings plus our earlier DCCS findings provide strong evidence against prominent cognitive complexity, conditional reasoning, and graded memory theories for why 3-year-olds fail these two tasks. The ways we have traditionally queried children may have obscured the budding reasoning competencies present at 3 years of age.

## Introduction

In conditional discrimination (CD) tasks with children (Gollin and Liss, 1962; Gollin, 1965; Andrews et al., 2012), which response is correct is conditional on which of two colors is present: Shape A is correct when Color 1 is present and Shape B is correct when Color 2 is present. Psychologists have assumed for over 50 years that the ability to do conditional, if-then reasoning does not develop until roughly 4 to 5 years of age, since children younger than that have consistently failed CD (Gollin and Liss, 1962; Gollin, 1965; Halford et al., 1998a; Halford et al., 2010; Andrews et al., 2012). We show here, however, that children barely 3 years old can succeed at CD (that is, are capable of conditional, if-then reasoning) when superficial stimulus properties are modified. To succeed at a CD task, a child must integrate the two dimensions (color and shape), yet psychologists have traditionally presented CD tasks as if the two dimensions were completely separate. Instead of making color a property of the background or of the outside border on stimulus cards (as in all previous CD experiments with children), we made color a property of the stimuli themselves (the shapes were either Color 1 or Color 2). This manipulation allowed children of 3 years to succeed.

Card sorting (as in the Dimension Change Card Sort [DCCS] task), on the other hand, requires attending only to color information when that dimension is relevant (ignoring shape) or attending only to shape information when that dimension is relevant (ignoring color) and being able to switch from doing one to the other. Correct sorting thus requires a child to separate the same two dimensions that CD requires a child to integrate. Depending on which dimension (shape or color) is currently relevant for sorting the cards, children are supposed to ignore the other. Until we (Diamond et al., 2005) and Kloo and Perner (2005) came along, psychologists had always presented card sorting tasks to children with both color and shape as properties of each stimulus object (e.g., a blue star or a red truck drawn on a stimulus card). Based on the repeated failure of children younger than $4+\frac{1}{2}$ to 5 years on the DCCS task, many had concluded that children younger than $4+\frac{1}{2}$ years are not capable of conditional reasoning or grasping a hierarchical rule structure (e.g., Frye et al., 1996; Zelazo et al., 2003; Andrews et al., 2012). We hypothesized that if color was a property of the background instead of a property of the stimulus as in canonical (or standard) DCCS testing that children would be able to successfully switch sorting dimensions at a younger age, and indeed that is what we found (Diamond et al., 2005). That simple manipulation enabled children to succeed on the DCCS test at 3 years – 12–18 months earlier than previously reported. Thus separating color and shape in the visual display aided 3-year-olds in conceptually ignoring one dimension when the task required that they focus on the other.

Both CD and DCCS require if, then conditional reasoning and they are both tasks that in their canonical forms 3-year-olds fail but children of 4–5 years pass. They are quite different tasks, however. For example, the rule structure for CD is that when Color 1 is present, Shape A is correct, and when Color 2 is present, Shape B is correct. The rule structure for DCCS is that when the sorting dimension is color, sort the stimulus card into the bin displaying the same color (ignoring that the shape on the stimulus card and target card over the bin do not match), but when the sorting dimension is shape, sort the stimulus card into the bin displaying the same shape (ignoring that the color on the stimulus card and on the target card do not match). Children are taught the rules for DCCS but not for CD. For CD, children must deduce the rules based on feedback. Feedback is provided on each CD trial but not on any DCCS trial.

We are not hypothesizing that these two tasks require all the same abilities or are in any way isomorphic. We are simply hypothesizing that on these tasks 3-year-olds can be strongly influenced by, and can be heavily dependent on, superficial, surface perceptual features of the stimuli. They can be helped to succeed by changing how things look.

Previously we asked ourselves, “Given the requirements of DCCS what surface modifications to the stimuli might help 3-year-olds?” It seemed to us that since children need to ignore one stimulus dimension when focusing on the other, it would be easier to do that if the two dimensions were not part of the same object (e.g., a drawing of a truck). Also, to the extent that for 3-year-olds a truck is either a truck or it is a red thing, but it cannot be both (Flavell et al., 1986; Perner and Lang, 2002; Kloo and Perner, 2003) separating the two dimensions so color is not an attribute of the truck should be helpful. It turned out we were correct; separating the dimensions did make the task easier for children of 3 and $3+\frac{1}{2}$ years (Diamond et al., 2005; Kloo and Perner, 2005).

Similarly, for the present study we asked ourselves, “Given the requirements of CD what surface modifications to the stimuli might help 3-year-olds?” The task requirements are different here than for DCCS. What is needed here is integrating the two dimensions of color and shape. What might help that? We reasoned that integrating them in the stimulus objects themselves should help. Any environment contains lots of perceptual information; how do children know what is relevant and what is not and what to attend to? Having color as an attribute of the truck and star drawings (integrated dimensions) should, we hypothesized, help children realize that color is relevant to the task. Since changing whether color and shape were integrated or separated improved the performance of 3-year-olds on DCCS to roughly the level of 4-year-olds, we hypothesized that changing whether color and shape were integrated or separated would improve the performance of 3-year-olds on CD so that it would roughly approximate the level of 4-year-olds.

Thus, our hypothesis here is that integrating color and shape in the visual display when the task requires conceptually integrating those dimensions (as does CD) should enable 3-year-olds to succeed because it bootstraps the children perceptually in their task of conceptually relating the two dimensions to one another. We tested this and present here the first demonstration that the age of first success on CD can be reduced from 4 or 5 years to 3 years by a surface modification of the stimuli. When color is a property of the stimulus object (i.e., color and shape are integrated as properties of the same object), instead of color appearing as part of the background (separated dimensions), 3-year-olds can succeed at CD.

We broke down our hypothesis into a set of predictions. For all predictions, the block of interest is Block 3. The reason for that is that Blocks 1 and 2 do not require conditioning reasoning. On Block 1 of our CD task, all cards contain blue and the reward is always hidden under the card with a truck drawing (the side of cards being pseudo-randomly varied across trials in all blocks). For Block 2, the reward is always hidden under the card with a drawing of a star, and all the cards contain red. On Block 3, cards containing blue and cards containing red are randomly intermixed over trials, though on each trial both cards contain red or both contained blue. The truck is correct when both cards contain blue (as in Block 1) and the star is correct when both cards contain red (as in Block 2).

Our predictions were:

(1) First and foremost, children of 3 years would succeed on CD (i.e., succeed on Block 3) when the dimensions of color and shape were integrated in the stimuli. Since we predicted that children of 3 years would perform roughly as well on CD (i.e., roughly as well on Block 3) with integrated dimensions as children of 4 years perform on CD with separated dimensions, we defined success on CD as roughly comparable Block 3 performance by 3-year-olds on integrated dimensions as 4-year-olds show on separated dimensions.
(2) Children of 3 years would perform significantly better on CD (i.e., on Block 3) when color and shape were integrated in the stimuli than when they were separated.
(3) We would replicate previous findings (e.g., Gollin and Liss, 1962; Gollin, 1965) that:
  (a) Children of 3 years will fail CD (i.e., fail to reach criterion in Block 3) when color and shape are separated on the stimulus cards (separated dimensions).
  (b) Children of 4 years will succeed on CD (i.e., succeed on Block 3) with that same condition (separated dimensions), i.e., they will perform roughly comparably on our CD task with separated dimensions to how other labs (Gollin and Liss, 1962; Gollin, 1965) have found 4-year-olds to perform on CD with separated dimensions when they tested that.
  (c) In all conditions and at both ages children would succeed on Blocks 1 and 2.

## Materials and Methods

### Participants

A total of 42 children were tested. All children could understand and converse in English and had normal or normal-with-correction hearing and sight. None were taking any medication that affected cognition. None had suffered a concussion or lost consciousness from a fall or blunt trauma to the head. This study was approved by the UBC Behavioral Research Ethics Board (REB# H04-80913), Vancouver Coastal Health Research Institute (V12-80913), and the Vancouver School Board. A parent or guardian of each child gave written informed consent for the child’s participation.

Participants were recruited from two age groups: children almost or just barely 3 years old and children almost or just barely 4 years old. They were tested in a StrongStart Centre in the greater Vancouver area (25 children) or in our lab at UBC (17 children). All children were accompanied by a parent, grandparent, or caregiver. The adult chaperone sat behind the child during testing or watched through the lab’s one-way mirror. A random subset of sessions was videotaped with permission from the parent or caregiver.

Six children (5 girls and 1 boy) were excluded from data analyses because they appeared unable to grasp how the task worked (that they were to retrieve rewards) or were not interested in it. Five of these children were 3 years old (3 tested on integrated, 2 on separated dimensions [including the one boy]). The sixth child was a 4-year-old girl tested on separated dimensions.

Our data set thus consists of 36 children; 22% were Caucasian, 22% East Asian, 8% Hispanic, 6% South Asian, 11% Mixed Ethnicity, and 6% were other, and 25% did not report their ethnicity. Most children (75%) came from a home where the primary caregiver has a college degree.

In the 3-year-old age group, there were 24 children (44% female). Half were tested on CD with integrated dimensions and half with separated dimensions. The mean age for the 3-year-olds was 3.1 years (SD = 0.16 years; range = 33.5–39.5 months). See Table 1.

**TABLE 1:** Age, number, and sex of children in each group.

**Condition**	**Age range**	**Mean age**	**SD age**	**#Male**	**#Female**	***N***
Integrated	2.8–3.3 years	3.07 years	0.16	6	6	12
Separated	2.8–3.3 years	3.05 years	0.16	6	6	12
Separated	3.8–4.4 years	4.03 years	0.22	9	3	12

In the 4-year-old age group, there were 12 children (33% female). They were tested on separated dimensions to see if, when we used the same procedure as have previous studies from other labs, we would get the same results. The mean age for the 4-year-olds was 4.0 years (SD = 0.22 years; range = 45.5–53.0 months). See Table 1.

*A priori* power analyses using G^∗^Power 3.1.9.2 (Faul et al., 2007) indicated that a total of 34 participants (11–12 per group) would provide 80% power to detect a medium effect size of 0.35.

### Materials

**FIGURE 1:**
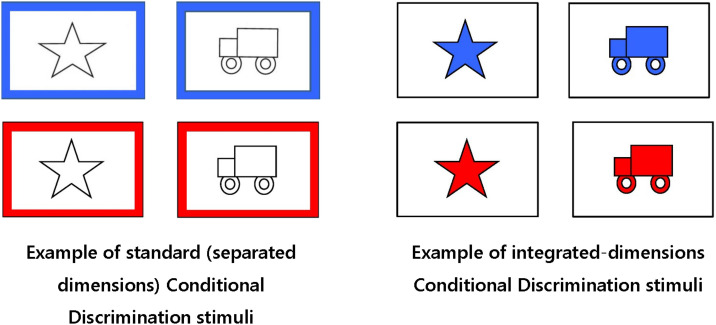
Examples of Standard (Separated Dimensions) and Integrated-Dimensions Conditional Discrimination Stimuli used in this study.

A child sat directly across from the experimenter at a table measuring 76 × 76 × 55 cm. The child was seated in a child-sized chair (36 × 30 × 36 cm) and the experimenter was seated on a stool (20 × 39 × 23 cm). Two rectangular wooden boxes open at the top, each measuring 12.5 × 8.6 × 3.7 cm, served as the containers where the reward was hidden. These boxes were identical in appearance. At the base of one of the boxes on the inside was a marble well. The marble well held the marble in place to prevent the child from guessing the marble’s location based on the sound of the marble rolling around. The stimulus cards served as the boxes’ lids. The 12 cards for each condition measured 13.3 × 9.6 cm each and were laminated. For the integrated condition, the 12 stimulus cards displayed a star or a truck that was either blue or red on a white background. For the separated condition, the shapes (star or truck) were white outlined in black and the border of the cards was either blue or red. See Figure 1.

**FIGURE 2:**
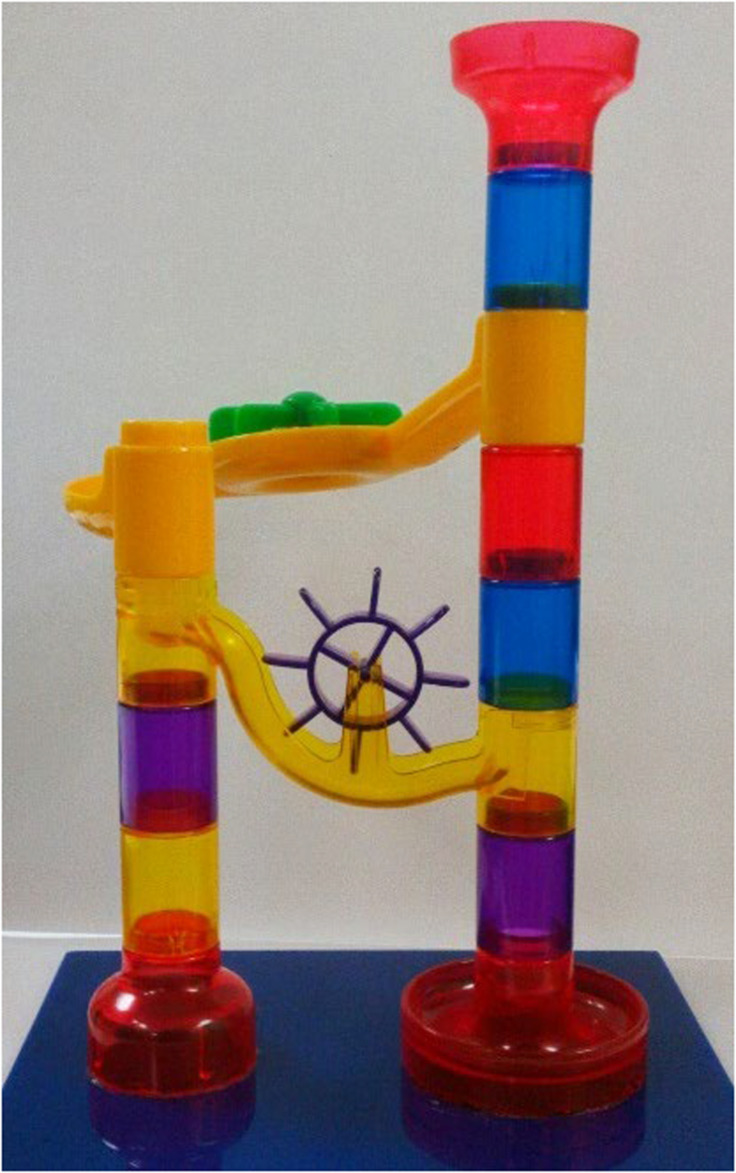
Marble maze used in the study.

Marbles (1.5 cm diameter) of different colors and patterns served as the reward. When children found a marble, they could put it in our marble maze and watch as the marble soared down ramps and spun through turnstiles. The marble maze (see Figure 2) stood 28.0 cm tall and was on a flat platform measuring 20.0 × 15.5 cm. A plastic, transparent jar (6.5 × 6.5 × 11 cm) was used to display the trove of marbles a child had found.

### Testing Procedure

First, the experimenter showed the child where to sit. The parent/guardian was given the option of sitting directly behind the child or watching from outside the testing room through a one-way mirror.

At the outset of testing, the experimenter told the child they were going to play a game and asked the child to cover his or her eyes like in the game “*Peekaboo*” (“*I am going to bring out a surprise. Can you cover your eyes, like this?*” [the experimenter showed child]). While the child’s eyes were closed and covered, the experimenter placed a marble in the marble well inside one of two boxes; this was the done underneath the table, out of sight even to the parent. Then the experimenter covered each box with a stimulus card (one showing a truck, the other showing a star) and placed the two boxes on the table, one to the left and one to the right, both equidistant from the child, and within the child’s reach. The left-right locations of the correct stimulus card was varied in the same pseudo-random sequence for all sessions (see below). The child sat, eyes covered, waiting in anticipation. The experimenter then announced: “*You can open your eyes now. I have hidden a surprise for you under one of these cards. Can you guess which one?*”

The child was encouraged to choose a card and lift it to see if the surprise was hidden beneath. If a child chose the correct card, the experimenter cheered exuberantly and encouraged the child to retrieve the hidden marble. The child was then presented with the marble maze and shown how to place the marble in it, to the enormous delight of the child.

If the child chose the wrong card, the experimenter said disappointedly, “*Oh no, it wasn’t there.*” The experimenter then lifted the correct card and showed the child where the reward had been hidden and said, “*It was here, see? Let’s try again. You’ll find it next time!*” The child was thus given feedback on each trial and the experimenter either cheered happily or showed the child what the correct choice had been and encouraged the child to find the marble next time. At no point did the experimenter ever explicitly state that the marble was under the truck or star or state the conditional rule. In Block 1, all the cards contained blue. The marble was always hidden under the truck card. The right-left location of the stimulus cards was pseudo-randomly varied across trials (Truck: Left, L, Right, L, R, R, L, R, L, L, R, R – repeated as long as needed up to a maximum of 36 trials). Participants never saw the reward being hidden and were never explicitly told the rules of the game. To find the reward they had to deduce the rule governing where it would be. As the right (R) and left (L) locations of the stimuli were randomly varied, always reaching right or left as a strategy did not lead to success. Six consecutively correct trials were required to pass the block and move on to the next one.

In Block 2, all cards contained red. Here, the marble reward was always hidden under the star stimulus card. Again, the right-left location of the stimulus cards was pseudo-randomly varied (Star: L, R, R, L, R, L, L, R, L, R, R, L – repeated up to a total of 3 times [36 trials]). The child needed to pick correctly on six trials in a row to pass criterion and move on to the final block.

On the first and second block, a child did not need to pay attention to color to choose correctly. The truck was always the correct choice for Block 1 and the star was always the correct choice for Block 2. There was no need to integrate color and shape information; attending to shape alone was sufficient.

In Block 3, cards containing blue or red were randomly intermixed over trials. On any given trial, both cards either contained red or blue. Again, the truck was the correct choice when both cards contained blue and the star was correct when both cards contained red. The following pseudo-random order indicates which color was presented on which trial and whether the correct choice was presented on the right or left: Blue + Left, Red + Right, Red + Left, Blue + Left, Red + Right, Blue + Right, Blue + Right, Red + Left, Blue + Right, Red + Right, Blue + Left, Red + Left. This was repeated as long as needed up to a maximum of 36 trials. As with Blocks 1 and 2, the criterion for passing Block 3 was six correct trials in a row.

The criterion for passing a block was 6 correct responses in a row within 18 trials. The choice of 18 trials was based on the work of Gollin and Liss (1962), who used 16–20 as their cut-off for Block 3 in their CD testing, after which the experimenter stepped in to aid the child in picking the correct stimulus. For each age X task group, we analyzed the number of trials needed to succeed on 6 trials in a row as well as the percentage of children who did so in 18 trials or less. We let children continue to try to figure out the CD rule after 18 trials, but only considered a child as having succeeded on a block if 6 correct trials in a row occurred within 18 trials or less.

Experimenter 2 (CDW) was blind to our hypothesis and predictions while testing the children. Experimenter 1 (DSL) was not blind to our hypothesis. Videos were taken of a random subset of those sessions where a parent gave permission (about 30% of sessions). The videos were reviewed by the senior author (AD) to check that children were treated comparably in both conditions and by both testers. Each experimenter also viewed the others’ tapes. AD noticed differences during practice and corrected them and would not approve the testers for testing until she was fully satisfied that they were doing each detail correctly and comparably. CDW and DSL each tested 50% of the children of 3 years in each condition. For children 4 years of age, DSL conducted 67% of the testing and CDW 33%.

## Results

Tester was not significantly related to any dependent variable for any test of our hypotheses, nor were gender or tester X gender, so those variables were dropped from further statistical tests. Analysis of variance (ANOVA) was used to test all hypotheses except for hypotheses concerning the binary variable of pass/fail, for which Fisher’s exact test was used.

**FIGURE 3:**
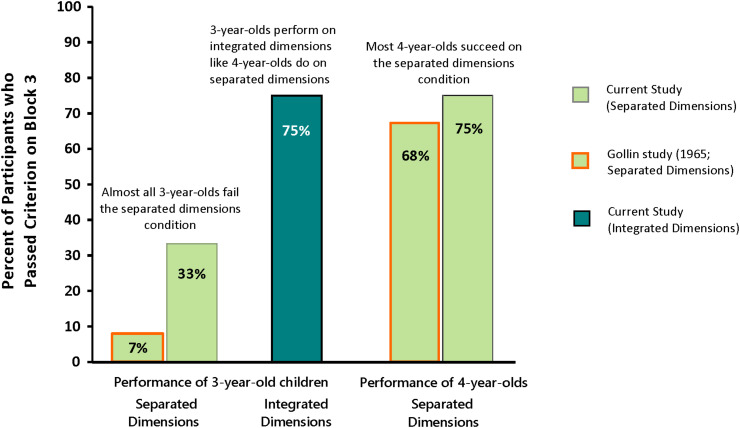
Percentage of Children 3 and 4 years old who Succeeded on Block 3 of the Conditional Discrimination Task in our study and in Gollin’s (1965) study.

**FIGURE 4:**
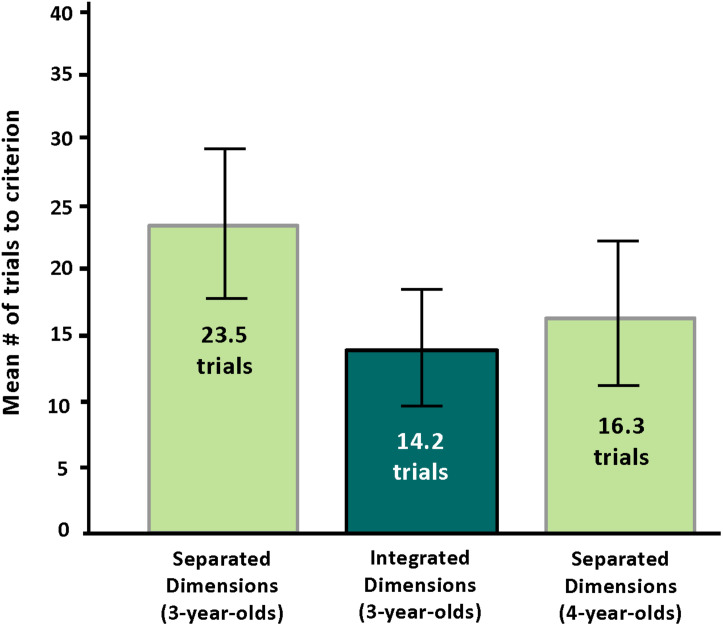
Mean Number of Trials to Criterion on Block 3 of Conditional Discrimination by Age and Condition in the present study.

**Prediction 1:** We turn first to our principal prediction, that children only 3 years old would succeed on CD with integrated dimensions. We operationalized “success” as performance by 3-year-olds on CD with integrated dimensions that is comparable to 4-year-olds on CD with separated dimensions. The percentage of 3-year-olds passing Block 3 of the integrated condition of CD (75%) was identical to the percentage of 4-year-olds passing Block 3 of the canonical version of the task (i.e., separated dimensions: 75%). See Figure 3. Children of 3 years took an average of 14.2 trials (SD = 7.8) to pass Block 3 when color and shape were integrated in the stimuli. Children of 4 years took an average of 16.3 trials (SD = 9.5) to succeed on the standard CD task when color and shape were separated in the stimuli. The number of trials needed to pass Block 3 was not significantly different between the two groups [*F*(1,22) = 0.36, NS]. Indeed, if anything, the number of trials was slightly lower for 3-year-olds with integrated dimensions than for 4-year-olds with separated dimensions. See Figure 4. We conclude that Prediction 1 was confirmed.

**Prediction 2:** We predicted that 3-year-olds would perform better on the integrated-dimensions condition than on the separated-dimensions condition. The percentage of 3-year-olds passing the integrated condition of CD (75%) was greater than the percentage of 3-year-olds passing the separated condition of CD (33%; Fisher’s Exact Test, *p* = 0.05). See Figure 3 above. Children of 3 years tested with separated dimensions (where color was a property of the border) took an average of 23.5 trials (SD = 9.8) to pass Block 3, whereas those tested with integrated dimensions (where color was a property of the truck or star) took an average of only 14.2 trials (SD = 7.8) to pass Block 3. That difference is significant: *F*(1,22) = 6.71, *p* < 0.02 (effect size: 0.23). See Figure 4. We conclude that Prediction 2 was confirmed.

**Prediction 3a:** We had predicted that we would replicate findings (Gollin, 1965) that 3-year-olds tested on CD with separated dimensions fail. Only 33% of the 3-year-olds tested with separated dimensions passed Block 3. Thus, most children of 3 years tested with the canonical version of CD failed (67%).

**Prediction 3b:** We predicted that when presented with the canonical CD task with separated dimensions, we would replicate previous findings (Gollin, 1965) that 4-year-olds succeed. Our results show that 75% of 4-year-olds tested on CD with separated dimensions succeeded. This is similar to what Gollin (1965) found, which was that 68% of 4-year-olds succeeded. See Figure 3.

**Prediction 3c:** Lastly, we predicted that in all conditions and at both ages, children would succeed on Blocks 1 and 2 as these blocks are fairly easy. In both Blocks 1 and 2, color was irrelevant because on all trials both cards contained the same color (Block 1: Blue; Block 2: Red). Indeed, all children tested, regardless of age or condition, passed Blocks 1 and 2. Children of 3 years tested in the integrated condition took 10.2 and 13.3 trials respectively to pass Blocks 1 and 2. Children of 3 years tested in the canonical separated-dimensions condition took 12.5 and 16.0 trials respectively to pass Blocks 1 and 2. Children of 4 years tested in the canonical separated-dimensions condition took an average of 10.3 and 11.2 trials respectively to pass Blocks 1 and 2. We conclude that all three sub-components of Prediction 3 were confirmed.

As an aside, when Block 1 begins children have no idea which shape is correct. When Block 2 begins children have had experience over 10–12 trials on average where the truck was always the correct choice and the star was never correct. On Block 2 that reverses, now the star always indicates where the reward is hidden and the truck never does. Since Block 2 requires a reversal, we expected that it would take children a bit longer to perform consistently correctly on Block 2 than Block 1. It did take children slightly longer, but the difference in the number of trials to criterion in Blocks 1 and 2 was never significant in either condition or at either age: for 3-year-olds on separated dimensions: *F*(1,10) = 1.19, NS; for 3-year-olds on integrated dimensions: *F*(1,10) = 2.98, NS; for 4-year-olds on separated dimensions: *F*(1,10) = 0.42, NS. These results are controlling for gender; without controlling for gender the *F*-values are even lower.

Our results provide evidence that integrating the dimensions allows children to perform CD at a level roughly 12–18 months ahead of when most had previously thought possible. Like others before us, we found that 3-year-olds fail and 4-year-olds succeed at CD presented the canonical way with color and shape separated on the stimulus cards (Gollin and Liss, 1962; Gollin, 1965; Rudy et al., 1993; Andrews et al., 2012).

## Discussion

We hypothesized that children of only 3 years are capable of conditional, if-then reasoning, but they do not seem to mentally integrate dimensions that are not properties of the same object. That is, when performing the CD task, they do not appreciate that the color in the background is telling them anything about which shape is the correct choice. On CD tasks children need to use the color shown to inform them which shape is correct (i.e., it is critical that they integrate color and shape information). We report here that when the dimensions of color and shape are integrated as part of the same stimulus, children of 3 years (12–18 months younger than previously reported) can use the value on one dimension (color) to indicate which value of the other dimension (shape) is correct, and thus succeed at CD. That is, they can deduce that red means the star is correct and blue that the truck is correct. When performing any task, one thing participants must do is determine which information in the environment is relevant and which is not. When color and shape are separated on the stimulus cards, 3-year-olds do not seem to comprehend that color is telling them anything about which shape is correct.

The current finding may be thought of as the flip-side of what we (Diamond et al., 2005) and Kloo and Perner (2005) found with the DCCS task. Children under 4 or 5 years typically fail to switch dimensions on DCCS. We hypothesized that if color and shape were not part of the same object, but instead if colorless shapes (black or white) were presented on cards with a background color, that children only 3 years old would be able to switch from sorting by color to sorting by shape or vice versa. Our hypothesis was confirmed (Diamond et al., 2005) and soon thereafter Kloo and Perner showed the same thing with colorless shapes and a color patch on each card. Children of 3 years can switch sorting dimensions when the dimensions are perceptually separate and not part of the same object.

The present findings together with those just cited for DCCS present the strongest evidence to date against several of the most prominent theories proposed for why 3-year-olds fail CD or DCCS. Evidently, 3-year-olds can grasp the hierarchical rule structure of the task (unlike Zelazo’s influential Cognitive Complexity and Control – Revised [CCC-R] hypothesis; Zelazo et al., 2003), have sufficient memory (unlike Munakata’s influential graded memory hypothesis; Morton and Munakata, 2002), and are capable of conditional, if-then reasoning (unlike Halford’s influential hypothesis; Halford et al., 1998b; Halford et al., 2010; Andrews et al., 2012) since when superficial stimulus properties were changed, children of 3 years succeeded.

The perceptual bootstrap we provided through changing superficial properties of the stimulus cards removed neither the need to grasp the embedded hierarchical rule structure, the memory demands of the task, nor the need for conditional “if, then” reasoning. Our study therefore suggests that scaffolding preschoolers’ emerging conceptual skills by changing the way the stimuli look (perceptual bootstrapping) enables 3-year-olds to demonstrate if-then conceptual reasoning abilities long thought beyond their grasp.

An alternative interpretation for our findings might be that success in the integrated-dimensions version of CD is due to simple associative learning, not conditional reasoning. Rather than learning that when the stimuli are blue, the truck is the correct choice, and when the stimuli are red, the star is correct, children might instead learn that “blue-truck” is correct and “red-star” is correct. Therefore, in Block 3 they simply scan for those two stimuli, and finding either, select it. An associative-learning interpretation, however, would have difficulty accounting for the findings that 3-year-olds needed an average of 8 trials and 4-year-olds needed an average of 10 trials before they started to consistently perform correctly in Block 3 (i.e., before their string of 6 correct responses in a row began). If it were simple associative learning, why did they need so many trials in Block 3? The first time, and every time, they saw a blue truck or red star they should have reached for that stimulus. Children should have been able to be consistently correct starting on Trial 1 of Block 3. Also, 2-year-olds, who are fully capable of associative learning, do not succeed at conditional discrimination, even with integrated dimensions. That is inconsistent with an associative-learning interpretation.

A more plausible alternative interpretation is kind of a linguistic interpretation. Perhaps children encode integrated stimuli as one word, e.g., red-star or blue-truck, like San-Francisco or South-Africa. Thus there is only one thing to remember for each condition. When the dimensions are separated, however, if the children even notice the color present in Blocks 1 and 2, they would need to hold 2 things in mind for each condition: star + red or truck + blue. Perhaps the latter puts too great a demand on their working memory. We cannot rule that out at present, though this explanation would not be applicable to the findings with DCCS.

**FIGURE 5:**
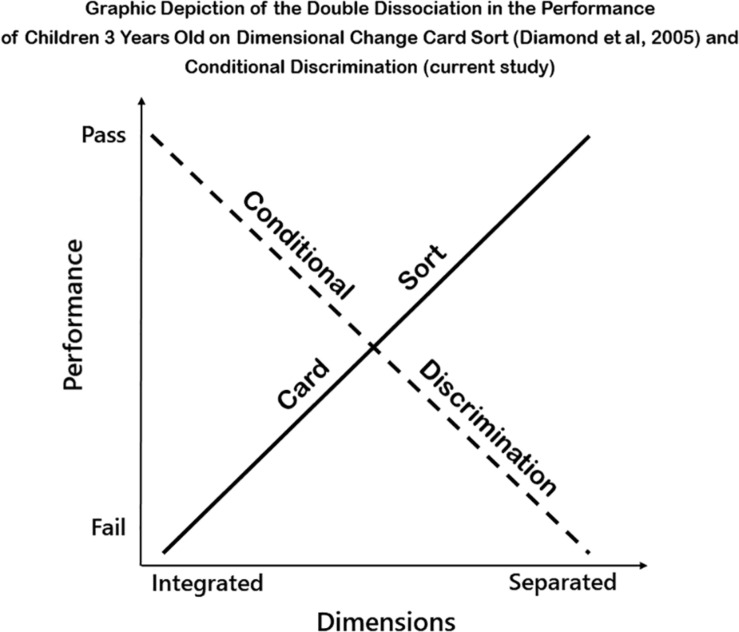
Graphic depiction of the double dissociation in the performance of children 3 years old on dimensional change card sort (Diamond et al., 2005) and conditional discrimination (current study).

The results reported here for conditional discrimination combined with those of Diamond et al. (2005) and Kloo and Perner (2005) on DCCS present a clear double dissociation between how integrating or separating the dimensions of color and shape affect the performance of 3-year-olds. What helps performance on CD hinders performance on DCCS and what helps performance on DCCS hinders performance on CD. Figure 5 symbolically displays this dissociation.

While both the CD and DCCS tasks involve conditional reasoning, CD requires the integration of dimensions while DCCS requires the separation of those same two dimensions. Could it be that the way we have traditionally queried children has not made it possible for 3-year-olds to show evidence of their budding reasoning competencies? Perhaps what children need help with is in understanding which information in the environment is relevant and which is not.

We acknowledge that there are limitations to our study. Our sample sizes were small – 12 per group. Our results are clear, however, and our number of subjects sufficient to find significant results. The sample sizes used here are quite comparable to those used by Kloo and Perner (2005) for testing separated versus integrated dimensions on the DCCS task. In one of their experiments, conditions were tested between-subjects (as here) and they had 12 participants per group (as here). In another of their experiments, conditions were tested within-subject with only nine participants per group. Another limitation is that we tested only one task (CD). It would have been more elegant to test the same children on both CD and DCCS. It would also have been more elegant to test each 3-year-old on both versions of CD, but we learned in using that design with DCCS that there was spillover from the easier condition to the more difficult one when tested within-child (i.e., children did better on DCCS with integrated dimensions when that was tested second than when it was tested first; Diamond et al., 2005). Also, CD testing takes longer than DCCS testing; almost none of the children we tested would have sat through another session testing the other CD condition. Another limitation is that Tester 1 was not blind to our hypothesis or predictions, as she had helped design the study. It is thus possible that she might have subtly, unintentionally affected children’s performance. Tester 2, however, was blind to the study’s hypothesis and predictions during testing, and we found no effect of tester on any outcome variable and no significant interaction between tester and any variable. Finally, we only tested children of 4 years on the integrated dimensions version of CD. While this last point might look like a limitation, since others have shown that 4-year-olds succeed on the more difficult version of CD (separated dimensions) it seemed unnecessary to test 4-year-olds on the easier version of the task (integrated dimensions).

Our results are consistent with those of other studies that used other paradigms. Jarvik’s (1956) study shows perhaps the most astonishing evidence. Many studies had shown that it takes chimpanzees over 100 trials to learn a simple visual discrimination (e.g., choose the red or green stimulus) when the reward is just below the stimulus card in a shallow well. Jarvik varied whether the reward was hidden 0.1 cm below the stimulus card in a shallow well or whether it was taped to a depression in the underside of the stimulus card. He replicated the result that with the reward in the well just below the stimulus card it takes chimpanzees an average of 131 trials to learn a visual discrimination. However, Jarvik found that chimpanzees were able to learn visual discriminations in *only one trial* when the reward was attached to the underside of the stimulus.

Our lab has previously shown the importance of perceptual modifications in other studies. When rewards were physically connected to the stimulus objects (e.g., by Velcro or even a string some inches long), infants only 9–12 months old could successfully use the stimuli to guide them to learn a delayed non-matching rule (choose the stimulus that does not match the sample you were just previously shown; Diamond et al., 1999; Shutts et al., 2001). When the reward is not attached to the stimulus object, but in the well just below, as in the canonical delayed non-matching to sample task, toddlers cannot succeed until they are 18–21 months old (Diamond, 1990; Overman, 1990; Diamond et al., 1994).

Deloache’s lab has likewise found results consistent with this: They report that toddlers of 18–22 months are significantly more likely to retrieve a reward they saw hidden when it is hidden inside a piece of furniture than when it is hidden near the same piece of furniture (DeLoache and Brown, 1983). Toddlers of 21 months can find a hidden object if it is hidden inside one of four attractive containers but they cannot use those same attractive containers to inform them where to search when those containers are mounted on top of four identical plain boxes (DeLoache, 1986).

In conclusion, if children of 3 years can succeed at CD when color and shape are integrated as part of the stimulus, then they must be capable of if-then, conditional reasoning at some level. It does not appear to be their reasoning ability that is lacking but rather what seems lacking is their ability to appreciate what information is relevant. Children of 3 years seem to rely on perceptual information (physical characteristics of the stimuli) to guide them in appreciating that the value of one dimension (color) is informing them about which value of the other dimension (shape) is correct.

## Data Availability Statement

The raw data supporting the conclusions of this article will be made available by the authors, without undue reservation.

## Ethics Statement

The studies involving human participants were reviewed and approved by the Behavioural Research Ethics Board of UBC, Ethics Review Board of the Vancouver Coastal Health Research Institute, and Human Ethics Review Board of the Vancouver School Board. Written informed consent to participate in this study was provided by the participants’ legal guardian/parent.

## Author Contributions

DL actively contributed to all aspects of this study and manuscript preparation. CW contributed to recruiting and testing the participants, entering the data, and thinking about what the results meant. AD actively contributed to all aspects of this study and manuscript preparation, except testing participants. All authors contributed to the article and approved the submitted version.

## Conflict of Interest

The authors declare that the research was conducted in the absence of any commercial or financial relationships that could be construed as a potential conflict of interest.
